# Lipid-Enriched Biopolymer Films for Active Packaging: A Review of Structure, Properties, and Preservation Performance

**DOI:** 10.3390/polym18070870

**Published:** 2026-04-01

**Authors:** Bruna Moura Bastos, Janaína Oliveira Gonçalves, Mariano Michelon, Luiz Antonio de Almeida Pinto

**Affiliations:** 1 Industrial Technology Laboratory, School of Chemistry and Food, Federal University of Rio Grande—FURG, Italia Avenue, km 08, Rio Grande 96203-900, Brazil; brunabastos.furg@gmail.com (B.M.B.); michelonmariano@gmail.com (M.M.); dqmpinto@furg.br (L.A.d.A.P.); 2 Department of Civil and Environmental, Universidad de la Costa, Calle 58 #55-66, Barranquilla 080002, Colombia

**Keywords:** bioactive compounds, bioavailability, biodegradable, essential oils, lipids

## Abstract

Amid growing environmental concerns regarding the use of non-biodegradable plastic packaging and its potential emerging contaminants, such as microplastics, currently among the most pressing global challenges, researchers in the food sector are increasingly pursuing sustainable alternatives. In this context, various organic sources have been explored for the development of innovative biocompatible films. These films exhibit properties such as low water vapor permeability, transparency, and biodegradability, and have recently gained active functionalities. These enable the extension of the shelf life of packaged foods by controlling microbial activity and oxidative degradation. Lipid-based compounds derived from animal and plant sources—including phospholipids, essential oils, free fatty acids, and saturated and polyunsaturated fatty acids—have proven highly effective when incorporated into films, leading to significant physicochemical, mechanical, and microbiological improvements in both the films and the packaged products. Owing to their high hydrophobic capacity, these lipids markedly reduce water vapor permeability, which is crucial for extending the shelf life of high-moisture foods. Studies have shown that the incorporation of lipid compounds can increase film tensile strength by up to 37% and enhance antioxidant activity by over 75%. Moreover, many of these compounds exhibit antibacterial and antimicrobial activities, becoming active on the surface of food in contact. However, many bioactive compounds have poor dispersion in aqueous solutions, limiting their effectiveness in the final product. When encapsulated with the aid of a lipid fraction, the bioavailability of these compounds is improved, and their release can be effectively controlled. This review aims to consolidate recent research on the production of biopolymer films incorporating various types of lipid compounds, highlighting their enhancements and potential applications in active food packaging systems.

## 1. Introduction

Biopolymer films have emerged as versatile materials for producing various products, including packaging, pharmaceutical capsules, scaffolds, and gels, across the food, pharmaceutical, and medical sectors [1,2,3]. These films are particularly valued for their physicochemical properties, such as tensile strength, elongation, and water vapor permeability, which can match or even surpass those of synthetic polymers under specific formulations [4,5,6].

In recent years, growing interest in multifunctional and sustainable materials has driven significant advances in the development of biopolymer-based films, particularly for applications in active food packaging [7,8]. The search for more sustainable alternatives is crucial nowadays due to the environmental consequences associated with synthetic packaging, which is mainly derived from petroleum-based materials such as polyethylene, polypropylene, and polyvinyl chloride [9]. Synthetic packaging is not only derived from non-renewable resources but also poses significant environmental challenges due to its long decomposition time and difficulty in recycling [10]. In contrast, biopolymer films are biodegradable, biocompatible, and can be derived from renewable agro-industrial residues such as peels, bones, seeds, and bagasse, which are rich in biopolymeric components (amino acids, proteins, and polysaccharides) that support film formation [11,12,13,14,15,16]. Moreover, in recent years, the search for materials that are both functional and environmentally friendly has inspired new developments in biopolymer-based films, especially for use in active food packaging [17,18].

To enhance the performance of these films and meet the growing demand for food preservation, research has focused on incorporating functional compounds into the film matrix. Among these, lipid-based compounds stand out due to their ability to improve water vapor barrier properties, oxidative stability, and antimicrobial functionality, especially in high-humidity environments [19,20,21,22]. Lipids, including essential oils, waxes, phospholipids, and unsaturated fatty acids, contribute to both mechanical reinforcement and the incorporation of bioactive properties in biopolymer films, making them suitable candidates for the development of active packaging systems [23].

Essential oils have garnered significant attention, not only for their ability to act as plasticizers but also for their natural preservatives with proven antimicrobial properties, offering an attractive alternative to synthetic additives. According to Regulation No. 450/2009 of the European Commission, active packaging incorporates substances that prolong shelf life by slowly releasing active agents or interacting with the internal environment of the package [24]. However, a key challenge in using lipid compounds in biopolymer films is maintaining their stability and functionality during processing and storage. Volatility, poor water dispersion, and phase separation during film formation can limit the effectiveness of these compounds unless strategies such as encapsulation are adopted [2,25,26,27,28].

Encapsulation techniques such as nanoemulsions, Pickering emulsions, and liposomes have emerged as effective delivery systems to enhance the stability, distribution, and controlled release of lipid compounds in film-forming solutions [29]. These approaches have shown promising results in extending antimicrobial activity, improving mechanical strength, and reducing moisture permeability of active films [30,31,32,33]. In addition, emerging approaches involving smart nanomaterials and biomimetic stabilizers have expanded the functionality of active films, allowing for stimuli-responsive release mechanisms and more efficient barrier control. Recent studies have yielded promising advances in the application of Pickering emulsions for food packaging [34,35]. However, the authors affirmed that in the practical application of biopolymer-based films, there are still challenges, especially regarding long-term stability and large-scale production.

Therefore, this review aims to provide a comprehensive overview of the incorporation of lipid-based compounds into biodegradable biopolymer films, with a focus on alternative encapsulation methods and their impact on the properties of these films. In addition, it aims to verify the use of essential oils, fish oils, nanoemulsions, and Pickering emulsions in packaging systems. Bringing their advances and challenges regarding food preservation and sustainable solutions for the packaging industry.

## 2. Lipid-Loaded Biopolymer Films

Biopolymers are macromolecules composed of repeating monomeric units derived from natural or synthetic sources [36,37]. These macromolecules vary according to their origin, degradability, polymeric structure (linear, branched, or cross-linked), and chemical nature (e.g., polysaccharides, proteins, nucleic acids), as well as their thermal behavior, being classified as elastomers, thermoplastics, or thermosets [36,38].

Therefore, these characteristics are highly relevant in the development of biopolymeric films for food packaging applications. The choice of biopolymer, for instance, plays a crucial role: polysaccharide-based films (like starch and pectin) typically offer greater resistance to gas permeation but are more prone to moisture absorption. Protein-based films (like gelatin and zein) are also sensitive to humidity; however, they generally exhibit better mechanical strength and flexibility [39,40,41]. The most widely used biopolymers for film production include gelatin [42,43,44,45], chitosan [46,47,48,49] and starch [45,50,51,52] due to their film-forming properties, biodegradability, and abundance.

However, using these biopolymers in their natural state may not always produce films resistant to synthetic materials, because some, especially gelatin and chitosan, exhibit hydrophilic behavior, which can limit their effectiveness in applications requiring low moisture permeability. In such cases, chemical modifications, such as incorporating crosslinking agents or applying ionic, electrostatic, and deprotonation modifications, are necessary to enhance the water vapor barrier properties of the films [40,53]. Table 1 compares the effects of Pickering emulsion applications in biopolymer films, highlighting the nature of the biopolymer, encapsulated lipid compound, emulsification method, droplet size (DS), water vapor permeability (WVP), and tensile strength (TS).

Table 1 shows that the use of solid particles to stabilize emulsions can significantly improve the physical and functional properties of films, especially regarding mechanical strength, water vapor permeability, and the retention of different active compounds. In the study described by Zhu et al. [57], a conventional emulsion prepared with an ethanolic solution was used, resulting in up to a 30% increase in the film’s antioxidant activity. The emulsions presented an average droplet size between 7 and 8 µm. While compatible with interfacial stability, these larger droplets showed limitations in aqueous dispersion, which may affect the uniform distribution and controlled release of bioactive compounds.

In contrast, the study by Gumus et al. [54] used fish gelatin combined with red wine pomace and carrageenan to formulate Pickering emulsions stabilized by cellulose nanocrystals. The resulting films demonstrated significant improvements in barrier properties, decreased TS, and increased film thickness. Moreover, these films demonstrated antimicrobial activity against Staphylococcus aureus and Escherichia coli, in addition to exhibiting high antioxidant potential, which highlights the multifunctionality provided by this approach. These findings emphasize that smaller droplet sizes (0.28 µm) promote the formation of a denser and less porous matrix, enhancing the film’s overall functional performance.

Other examples using fish gelatin were described by Sánchez et al. [71]. The addition of *Aloe vera* significantly affected the mechanical properties (also increasing thickness) and led to a notable improvement in thermal stability and antimicrobial activity in the films, indicating that fish gelatin can be transformed into effective active packaging. Wu et al. [56] also observed similar results using cinnamon essential oil in fish gelatin films (*silver carp*, *Hypophthalmichthys molitrix*). The authors reported that the addition of the lipid compound reduced TS values, significantly increased film thickness and WVP, corroborating the findings of other studies in the literature.

Li et al. [72] developed films using fish gelatin (*silver carp*) and found that the incorporation of natural antioxidants, such as grape seed extract, compromised the mechanical properties of the film, with a significant decrease in both TS and elongation at break. Therefore, these results indicate that the chemical nature of the additive, the compatibility of the components, and especially the synergy between them in the final matrix directly influence the final characteristics of the developed film.

The principle of combining emulsions with nanocarriers is also reinforced in studies using nanocellulose fibers. Fahim et al. [73] and Ding et al. [74] highlight that emulsions combined with nanocellulose fibers, together with essential oils, improve the mechanical and water resistance of gelatin-chitosan films. The authors attribute the formation of a more cohesive polymeric network, which is capable of uniformly incorporating bioactive lipids and hindering their migration to the outside, therefore favoring the stability of the material and its performance as active packaging.

This correlation between droplet size and performance is a key point. Tang et al. [65] showed a high tensile strength (30.49 MPa) and an impressively low water vapor permeability of 1.88 × 10^−11^ kg·m/m^2^·s·Pa with a minimum droplet size of 3.15 µm. This correlation between smaller droplet size and enhanced barrier performance is further confirmed by Liu et al. [67], who obtained an even lower water vapor permeability of 1.68 × 10^−12^ g/m·s·Pa by utilizing a significantly smaller droplet size of approximately 0.15 µm.

The versatility of starch is also a factor to consider. Irikura et al. [53] highlighted how starch sourced from different botanical origins influences film strength and flexibility, reinforcing the versatility of starch in biodegradable film formulations. Zhang et al. [62], whose findings demonstrated that formulations with adjusted droplet size distributions, especially within smaller ranges, increase emulsion stability and the functional efficacy of the resulting films. Thus, droplet sizes below 1 µm tend to result in more stable emulsions, as they reduce the tendency for coalescence and ensure better dispersion within the polymeric matrix. Furthermore, this homogeneity contributes to improved performance in the controlled release of bioactive compounds, such as essential oils and antioxidants. Importantly, both Zhang et al. [62] and Du et al. [63] employed zein/pectin nanoparticles as stabilizers of Pickering emulsions. While Zhang et al. [62] focused on the effect of droplet size, Du et al. [63] advanced this approach by incorporating zein/pectin stabilized emulsions into Konjac Glucomannan-based films and applying ionic crosslinking with Ca^2+^, Cu^2+^, and Fe^3+^. This strategy enhanced the tensile strength from 3.62 MPa up to 12.49 MPa, improved water resistance, and conferred superior UV-blocking, antioxidant, and antimicrobial activities. These results demonstrate that combining zein/pectin systems with ionic crosslinking can significantly enhance the functional performance of biopolymer-based films for food packaging applications.

However, zein has also emerged as a promising biopolymer for use in active films, particularly in Pickering emulsions [75], due to its hydrophobic nature and disulfide bonds, which confer improved heat resistance, moisture tolerance, and abrasion resistance [62]. Studies have shown that incorporating zein into chitosan-based films significantly reduces water vapor and oxygen permeability, thereby enhancing the preservation of perishable foods, such as edible mushrooms [58].

### 2.1. Biopolymeric Films Added with Essential Oils

Essential oils are natural, volatile compounds rich in bioactive phenolic substances, widely recognized for their potent antimicrobial and antioxidant effects. These features make them ideal candidates for incorporation into biodegradable food packaging films, where they can inhibit microbial growth, delay oxidation, and extend product shelf life. From a mechanistic standpoint, the bioactivity of essential oils is primarily attributed to their ability to disrupt the integrity of bacterial cell membranes, interfere with enzymatic systems, and scavenge free radicals that promote lipid oxidation [18,76]. Therefore, technological alternatives have been studied to minimize these constraints, such as microencapsulation with suitable wall materials, coating with biopolymers, or even the synthesis of nanocomposites [18,76,77,78]. These technologies enhance the stability of essential oils, promote controlled release, and preserve their functionality over time, factors that are critical for ensuring consistent antimicrobial action throughout the film’s use.

Therefore, it is important to emphasize that essential oils influence mechanical properties, barrier function and also bioactivity, which is directly influenced by the concentration of the oil and compatibility with the polymer matrix. Higher concentrations of oil can increase and favor flexibility due to plasticization effects, but excessive amounts can impair the cohesion and integrity of the film.

Khruengsai et al. [62] developed chitosan films incorporating *Zanthoxylum limonella* essential oil at varying concentrations (0%, 2%, and 4%) and evaluated their antibacterial activity against *Escherichia coli* and *Staphylococcus aureus*. The study found that incorporating 4% essential oil significantly enhanced the antibacterial properties of the films, providing adequate protection against these bacteria compared to chitosan films without essential oil. Moreover, the addition of essential oil improved the mechanical strength and flexibility of the films while maintaining minimal changes in water solubility, water vapor permeability, and thermal stability. Marvizadeh et al. [79] presented significant antimicrobial activity, with inhibition zones of 90 mm in diameter against *E. coli* and 55 mm against *S. aureus*.

Chitosan-based matrices tend to exhibit greater compatibility with essential oils, resulting in superior antimicrobial and mechanical properties compared to starch-based formulations [30]. However, further advancements are still needed due to the high volatility and uneven dispersion of the oils, which remain key challenges. These issues often require more sophisticated encapsulation strategies and techniques, which will be discussed in Section 3.

**Figure 1:**
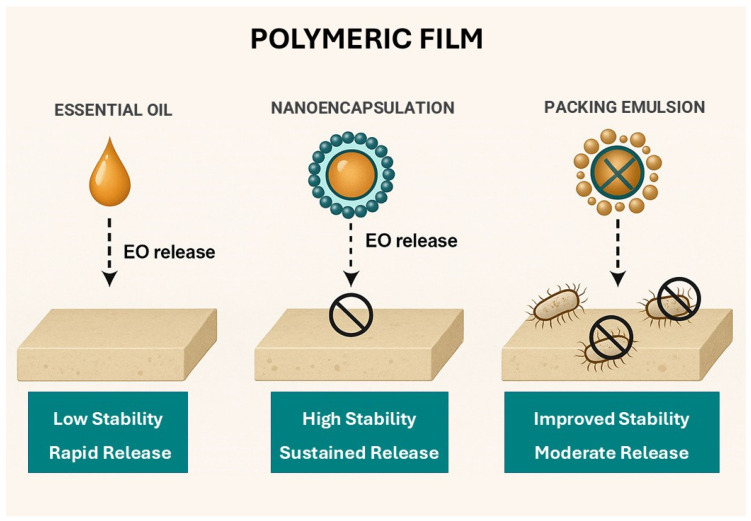
Schematic representation of a biopolymeric film incorporated with essential oil.

Figure 1 presents a representation of different approaches for incorporating essential oils, including direct addition, nanoencapsulation, and Pickering emulsions, and illustrates their respective impacts on release behavior, stability, and antimicrobial activity of the films. This visual summary reinforces that the choice of encapsulation method plays a fundamental role in optimizing the functionality of essential oils in active packaging systems.

Incorporating essential oils stands out as one of the most promising strategies for active packaging. However, successful incorporation depends not only on their bioactivity, but also on how effectively they are stabilized and retained within the film during storage and application. Another point to be highlighted is that the use of essential oils also brings their natural aroma to the films. While this can be beneficial in some applications, it may be unsuitable for foods with more neutral or delicate flavors. Oils like cinnamon (*Cinnamomum verum*), thyme (*Thymus vulgaris*), and peppermint are among the most commonly used due to their pleasant scent and antimicrobial activity; however, their intensity should be carefully balanced.

### 2.2. Biopolymeric Films Added with Fish Oils

Fish oils have been extensively produced, researched, and integrated into foods and daily diets due to their rich content of omega-3 fatty acids, which are long-chain polyunsaturated fatty acids (PUFA) with multiple carbon-carbon double bonds [80]. These fatty acids, including docosahexaenoic acid (DHA; 22:6n−3) and eicosapentaenoic acid (EPA; 20:5n−3), are essential nutrients that must be obtained through the diet, primarily from marine animals and seafood. The high concentration of omega-3s in fish oils offers numerous health benefits, including maintaining cell membrane integrity and enhancing cellular signaling and interactions, particularly in the heart, brain, and nervous system. As a result, fish oil has gained recognition as a functional food component [81,82].

Beyond their nutrition applications, fish oils have emerged as potent ingredients in the formulation of polymeric films, thanks to their inherent hydrophobicity, which improves water vapor barrier properties of these films [83,84].

Several studies have investigated the incorporation of fish oils into biopolymer matrices, aiming to improve their physicochemical properties. For instance, Bastos et al. [68] developed biopolymer films based on crosslinked gelatin from common carp skin, incorporating chitosan and bleached tuna oil or tuna-free fatty acids. Their results showed that all the films outperformed the control in terms of mechanical properties, with the formulation containing chitosan alone showing the best overall performance. Specifically, the crosslinked gelatin film with chitosan exhibited a tensile strength of 26.7 MPa, an elongation of 12.9%, and a water vapor permeability (WVP) of 1.37 × 10^−11^ g m^−1^ s^−1^ Pa^−1^. Conversely, the film enriched with both chitosan and free fatty acids exhibited a lower tensile strength (11.3 MPa) but markedly higher elongation (332.1%) and improved WVP (1.04 × 10^−11^ g m^−1^ s^−1^ Pa^−1^).

These findings highlight the dual impact of fish oils on film performance: while tensile strength may decrease, elasticity and water vapor resistance tend to increase. Scanning electron microscopy (SEM) corroborated these results by revealing encapsulated oil droplets dispersed throughout the matrix, contributing to enhanced flexibility and barrier properties. These outcomes are consistent with similar results reported by Ramziia et al. [85] and Ahmed and Ikram [86].

The evaluation of mechanical resistance and elongation at break is crucial in determining the suitability of these materials for food packaging. An ideal packaging material should strike a balance between strength and flexibility to withstand physical stress during handling. Additionally, a lower WVP reflects reduced moisture absorption, which helps preserve the food’s texture, flavor, and shelf life. Therefore, incorporating fish oils into biopolymer films represents a promising strategy for developing multifunctional and durable packaging solutions.

### 2.3. Conventional Emulsions in Films

Emulsions are colloidal systems formed by mixing two immiscible liquids, typically water and oil. This process leads to the formation of small spherical droplets (dispersed phase) suspended in another liquid (continuous phase), thereby greatly increasing the interfacial area of the system [87]. However, due to their thermodynamic instability, emulsions are prone to phenomena such as droplet coalescence, flocculation, and phase separation [88]. To ensure stability—especially in applications like food packaging where consistency is critical—the use of emulsifiers or stabilizing agents becomes essential [89].

In the context, emulsions have been widely investigated for encapsulating oils and other bioactive compounds within biopolymer films. This incorporation technique enables a uniform distribution of the active compounds within the film matrix, improving both their functional performance and their controlled release over time [90]. In addition, the incorporation of emulsions into biopolymer films also enhances their barrier properties, particularly against water vapor, owing to the hydrophobic characteristics of the emulsion’s oil phase [2].

Moreover, encapsulating bioactive compounds within emulsions before their integration into film-forming solutions offers significant advantages. It provides a controlled release mechanism, thereby prolonging the efficacy of the active ingredients and enhancing their protective role within the film matrix [91]. By stabilizing these compounds, emulsions contribute to a more efficient and targeted delivery system within biopolymer films, which is crucial for applications in food packaging and other fields where prolonged activity and stability are required [92].

**Figure 2:**
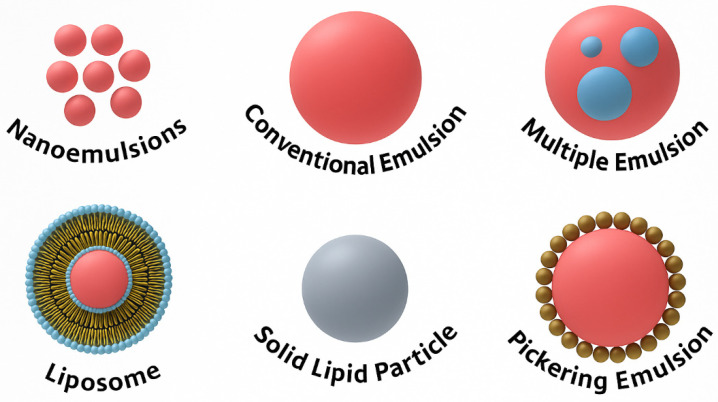
Advanced emulsion systems with food-grade ingredients.

The versatility of emulsion-based delivery systems has driven the development of a range of advanced emulsions tailored for specific applications. These include Pickering emulsions, double emulsions (W/O/W), and nanoemulsions, each offering unique advantages in terms of stability, release profile, and compatibility with sensitive bioactives. Figure 2 summarizes these key emulsion types and their features, illustrating the diversity of design possibilities for active packaging applications.

#### 2.3.1. Nanoemulsions in Active Films

Nanoemulsions are dispersed colloidal systems of two immiscible liquids, usually oil and water, into a stable mixture using emulsifiers and high-energy mechanical techniques [93]. These systems often exhibit droplet sizes below 100 nm, which are achieved through high-shear processing. This significantly increases the specific surface area and contributes to greater physical stability over time [94]. The emulsifier plays a crucial role by forming a thick interfacial layer around the droplets, minimizing density differences and thus reducing the risk of coalescence or phase separation [87,90].

In function of the nano size and structural stability, nanoemulsions tend to exhibit higher transparency, better droplet uniformity, and improved dispersion within polymer matrices when compared to conventional emulsions [95,96]. These attributes make them especially suitable for use in active biopolymeric films, where uniform distribution of bioactives and long-term stability are essential. Table 2 presents the mechanical and antimicrobial properties of biopolymer films to which different types of nanoemulsions were added.

The study by Feng et al. [82] is an excellent example of the importance of selecting the correct polymer matrix. The authors encapsulated cinnamon essential oil (*Cinnamomum verum*) in modified octenyl succinate anhydride (OSA) starch nanoemulsions to incorporate into pullulan-based films. These biodegradable films showed excellent performance in terms of barrier and mechanical properties, particularly when higher concentrations of cinnamon oil were used. The study indicated that the biodegradable films exhibited excellent performance in terms of mechanical properties (36.5%) and moisture barrier (0.056 g·m^−1^·kPa^−1^·h^−1^), especially when higher concentrations (5.3 g kg^−1^) of cinnamon oil were used, suggesting strong potential for food preservation. Pullulan has gained increasing attention in the literature due to its excellent gas barrier properties and its contribution to film-forming characteristics in biodegradable materials [97,98]. Shen et al. [99] developed composite films based on pullulan and gelatin incorporating Pickering emulsions stabilized with clove essential oil. Their findings showed that although the films containing Pickering emulsions exhibited moisture barriers and antioxidant properties comparable to those of conventional nanoemulsions, they promoted a significantly slower release of the active compound. This observation is particularly relevant as it demonstrates that the type of emulsion stabilization, Pickering vs. nanoemulsion, can directly influence the release profile of bioactive agents, a critical factor for the long-term functionality and performance of active packaging systems.

Another polysaccharide that also stands out is sodium alginate, which helps in the formation of a barrier against oxygen and is biodegradable. However, a barrier to moisture limits this, and studies show that the incorporation of cinnamon essential oil nanoemulsions significantly mitigates this problem [100,101,102]. For instance, Vieira et al. [83] incorporated olive oil nanoemulsions into chitosan/alginate matrices and showed reduced tensile strength (3.3 MPa and 5.0 MPa, respectively), and the pullulan–cinnamon oil films demonstrated superior mechanical integrity (up to 49.3 MPa). These findings reinforce that the type of nanoemulsion and its compatibility with the film-forming matrix are critical factors in achieving functional packaging properties [103,104,105].

Analyzing the data in Table 2, we can observe that the addition of lipid nanoemulsions generally increases the Elongation Strength (EL), indicating greater flexibility. Conversely, the Tensile Strength (TS) varies. The behavior of these films suggests a trade-off between stiffness and flexibility that must be optimized for the specific application. For example, gelatin film with black pepper essential oil [87] exhibits an exceptionally high TS (77.32 MPa) and a very low ELS (1.82%), making it ideal for packaging that requires stiffness, such as dry snacks. On the other hand, alginate films with lecithin and thymol [86] and, especially, gelatin [4] with the same components, with ELS above 100%, are more suitable for packaging products that require high flexibility, such as soft fruits.

Table 2 also reveals that different matrices can achieve similar antimicrobial efficacy. For example, pectin films with marjoram essential oil [84] and alginate films with lecithin and thymol [86] exhibit comparable inhibition zones against *E. coli* and *S. aureus*.

Films containing Opuntia oligacantha and orange oil [106], cinnamon essential oil [86], or black pepper essential oil [87] demonstrated relevant improvements in antioxidant and antimicrobial activities, although the mechanical properties varied considerably depending on the oil type and concentration. In particular, the black pepper essential oil films showed exceptionally high tensile strength but very low elongation, indicating that they are more suitable for rigid packaging applications. Other gelatin films with lecithin and thymol nanoemulsions [104] stood out for their high flexibility, with elongation values above 100%, making them more suitable for packaging soft and perishable foods.

The recent study by Xia et al. [107] adds an interesting perspective to these findings. Instead of incorporating a single lipid nanoemulsion, the authors designed a three-phase Pickering system where bacterial cellulose nanocrystals, carnosic acid, and polylysine worked together within the gelatin matrix. This formulation combined structural reinforcement, antioxidant protection, and antimicrobial activity in one film. What makes this approach particularly remarkable is not only the multifunctionality achieved but also its validation in real food packaging: when applied to cheese, the films extended shelf life while maintaining sensory quality. Compared to previous gelatin-based systems [108,109,110,111], which typically focus on improving either mechanical or antimicrobial performance, the author demonstrates the potential of Pickering emulsions to deliver more stable, multifunctional, and application-ready active films.

The study with calcium alginate films augmented with thyme essential oil [105] showed moderate improvements in mechanical properties (TS = 23.35 MPa) and antimicrobial effect confirmed by the protective halos against *E. coli* (8.4 mm) and *S. aureus* (8.6 mm). However, the relatively low elongation values (9.4%) indicate that these films present less flexibility, which may limit their application in products that require more malleable packaging. In contrast, Dong et al. [112] found that the addition of cinnamaldehyde nanoemulsions to chitosan/alginate trilayer films not only significantly increased tensile strength (15.0 to 25.5 MPa) but also improved elongation (17.5% to 23.5%). Furthermore, this latest study is highlighted to validate the performance of the films in the preservation of carp fillets, extending their shelf life by eight days.

Amjadi et al. [113] developed whey protein isolate films with orange peel essential oil nanoemulsions, which showed high elongation but very low tensile strength, suggesting that they are better suited for flexible rather than mechanically demanding packaging. In comparison, Ghadetaj et al. [114] demonstrated that the incorporation of *Grammosciadium ptrocarpum* essential oil nanoemulsions improved both tensile strength and elongation while also enhancing antioxidant and antimicrobial performance. This indicated that whey protein isolate can act not only as a film-forming agent but also as a stable matrix for bioactive delivery. Supporting these findings, Elahi et al. [115] applied a nanochitosan–whey protein isolate composite coating enriched with summer savory oil directly to rainbow trout fillets, in combination with oxygen absorbers, and showed strong antimicrobial activity in real food preservation. Taken together, these studies highlight the versatility of whey protein isolate: depending on the formulation, it can be tailored to improve flexibility, enhance barrier and bioactive properties, or even function effectively as part of complex preservation systems applied directly to foods.

Furthermore, nanoemulsion-based films often exhibit high encapsulation efficiencies (typically above 80%) and controlled release behavior, which are influenced by droplet size, emulsifier type, and interactions with the polymer matrix [86]. These characteristics enhance the long-term antimicrobial and barrier properties of the active packaging [106]. Based on these data and comparisons, it is essential to align the emulsion composition (lipid type and emulsifier) with the specific requirements of the packaging system.

**Table 2:** Films properties for active packaging based on natural polymers added with nanoemulsions of different origins.

Films	Nanoemulsion	Thinkness (mm)	TS (MPa)	EL (%)	Halo (mm)				
					*E. coli*	*S. aureus*	*A. niger*	*M. racemus*	References
Pectin	Marjoram essential oil	0.090	3.95	25.18	12.65	14.32	-	-	[105,113]
Calcium Alginate	Thyme essential oil	0.036	23.35	9.40	8.40	8.60	-	-	[65,105]
Alginate	Nanoemulsions with 0.5% soy lecithin and 0.5% thymol	0.049	23.40	21.40	12.50	11.16	-	-	[104,114,115]
Whey protein isolates	2.5% orange peel essential oil	0.230	0.81	38.57	7.67	-	-	-	[106,113,114,115]
Gelatin	Opuntia oligacantha C.F. Forst. and orange oil	-	21.00	18.00	-	-	-	-	[108,113]
Gelatin	black pepper essential oil	0.070	77.32	1.82	49.35	-	-	-	[103,109]
Gelatin 2.5%	Nanoemulsions with 0.5% soy lecithin and 0.5% thymol	0.087	5.22	104.50	14.84	-	-	-	[4,110]
Carboxymethylcellulose (CMC)	Cinnamon essential oil	0.126	6.05	94.97	-	-	15.50	20.00	[104]
Chitosan	Curcumin	-	7.96	54.07	-	-	-	-	[46,116]
Gelatin and chitosan	α-tocopherol and cinemaldehyde	0.081	11.40	108.70	-	-	-	-	[6,115]

TS: tensile strength; EL: elongation; Note: cinnamon oil (*Cinnamomum verum*); black pepper essential oil (*Piper nigrum*); orange peel essential oil (*Citrus sinensis*).

#### 2.3.2. Pickering Emulsions in Films and Packaging

The concept of Pickering emulsions dates to 1907, when Pickering published his article on stabilized emulsions formed by insoluble emulsifiers, such as basic iron and copper sulfates, which opened a new range of possibilities for studying emulsions and their applications [117,118,119]. Pickering oil-in-water (O/W) or water-in-oil (W/O) emulsions can be produced, depending on the relative affinity of the solid particles for one of the phases [120,121].

**Figure 3:**
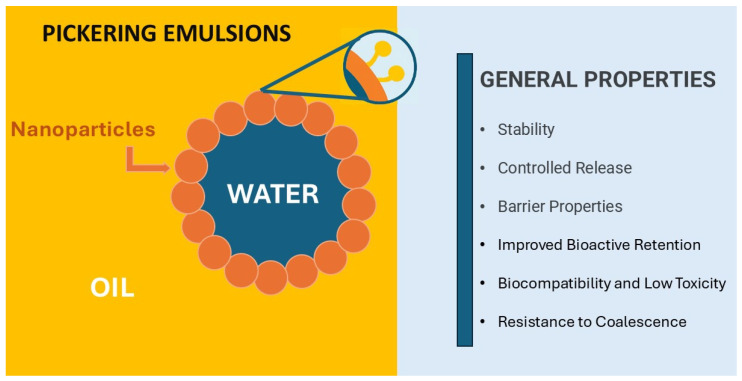
Pickering emulsions: scheme.

Figure 3 illustrates the general properties and mechanism of Pickering emulsions by which solid particles stabilize the interface between immiscible liquids.

Pickering emulsions are considered highly stable due to the adsorption of solid particles at the interface, which promotes the formation of an interfacial layer that sterically prevents emulsion destabilization [122]. Due to the high desorption energy, an energy barrier is formed associated with the attractive forces at the interface between the particles and the liquid phases [121]. As a result, the liquid interface around the particles undergoes deformation, which favors the formation of a more insurmountable interfacial layer that prevents the coalescence of droplets [123]. The desorption energy of the particle at the interface can be estimated by Equation (1), where γ is the oil/water interfacial tension and *a* is the particle radius [124].

(1) $$\varepsilon =\gamma \;\pi \;a^{2}\;\left(1-|\cos\theta \right|{)}^{2}$$

After emulsion formation and solvent removal, the droplets of Pickering emulsions provide empty structures, with adjustable pore sizes, which favor their use as a model to produce foam materials and in polymerization processes [121]. In addition to the advantage that solid particles are suitable stabilizers for this type of emulsion, many of these particles impart their functional characteristics, such as conductivity and porosity, to the emulsions (and consequently to the films). Also, many of them have food quality and lower toxicity, which leads to greater safety for in vivo use [120,125,126,127].

Chitin nanofibers and nanocrystals have been widely employed in stabilizing Pickering emulsions due to their versatile properties and ability to contribute to various functions of these emulsions [58,128]. Positively charged chitin nanocrystals are adsorbed at the oil-water interface to form a network in the continuous phase and between emulsion droplets, providing high stability to the resulting emulsions, which can be adjusted by modifying the pH and concentration [129]. On the other hand, chitin nanofibers, derived from the breakdown of chitin into fibrous structures, exhibit superior emulsifying properties to chitin nanocrystals, especially at higher concentrations [130,131].

Jiménez-Saelices et al. [104] used chitin nanocrystals to stabilize Pickering (o/w) emulsions with paraffin into a starch-based film. These were dispersed by up to 45%v in a starch-based film-forming matrix with lower than 1% nanocrystals. Microscopic analysis showed composite films containing droplets of Pickering emulsions with a diameter of 3–5 µm, homogeneously dispersed throughout the thickness of the films. In addition, mechanical analyses showed that the performance of the films was driven by the percentage of pure vitreous starch, remaining stable up to 32%v of emulsion added. Above this value, due to the greater presence of flocculated emulsion droplets, the starch matrix was discontinuous. As a result, points of weakness were identified, which negatively impacted both the film’s stiffness and its ability to resist stretching, and its percentage of maximum deformation. The smaller the size of the emulsion droplets added, the lower the possibility of coalescence, and the more homogeneous the film will be, which will guarantee a better dispersion and consequent delivery of the emulsified compound. Furthermore, the chemical structure of the hydrophobic compounds that comprise the emulsion contains groups capable of interacting with greater force with the polymeric chains of the biopolymers present in the film-forming solution. This enables the reduction in film mobility and enhancement of its elongation, thereby facilitating its use as a food packaging material [84,132].

Chemical modifications, using blending polymers or crosslinking agents, can enhance the mechanical properties of films containing Pickering emulsions [74]. Xie et al. [133], highlighted a significant improvement in tensile strength due to the crosslinking, providing a structural rearrangement and improving the interlocking between biopolymeric networks. Xu et al. [105] demonstrated that the blend of zein with chitosan in the formation of nanofibers facilitates compatibility between the film-forming matrix and the Pickering emulsion. The authors justify this due to the presence of strong crosslinking interactions and the formation of new hydrogen bonds, particularly in conjunction with essential oils.

Xu et al. [105] developed active packaging films using oregano essential oil (OEO) stabilized by TEMPO-oxidized chitin nanocrystals (TOCN), which were incorporated into the Konjac glucomannan (KGM) matrix to form an active packaging film. The OEO Pickering emulsions stabilized by TOCN, with 2.4% by weight of TOCN and 2% by volume of OEO, exhibited a uniform droplet distribution, with a particle size of 25.80 μm. The incorporation of the OEO Pickering emulsion compared to the film without addition reduced the tensile strength and elongation at break mechanical properties of the films. However, the water vapor permeability of the films increased when concentrations of 30% and 40% of the emulsion were added. As for the antioxidant and antimicrobial properties of the films, these were strengthened by the incorporation of the OEO Pickering emulsion, increasing with increasing concentration.

Cellulose-derived nanomaterials, such as nanofibers and nanocrystals, are highly promising as Pickering emulsion stabilizers. Being abundant, sustainable, and non-toxic, these materials replace conventional surfactants, making emulsions more eco-friendly [35,133,134]. They offer excellent thermal stability, particle dispersion, and storage life across various essential oil systems [135,136,137,138,139,140].

Table 3 provides an overview of the reviewed studies, highlighting the type of biopolymer used and the added lipid fraction using Pickering emulsions.

From Table 3, it was observed that the main results of studies exploring the importance of Pickering emulsions in the development of biopolymer-based films incorporated with different lipid fractions. The use of soy protein isolate (SPI) in oxidized corn starch films resulted in an increase in elongation at break from 57% to 150.93% and a decrease in WVP from 3.30 to 2.34 × 10^−12^ g·cm·cm^−2^·s^−1^·Pa^−1^, demonstrating the synergistic enhancement of flexibility and moisture barrier properties [136].

When the objective is to incorporate more sensitive active compounds, such as essential oils, the strategy is enhanced by using Pickering emulsions. In emulsions stabilized with pistachio shell cellulose nanocrystals, a reduction in droplet size from ~17 µm to ~2 µm was achieved as cellulose nanocrystal concentration increased, promoting higher emulsion stability and better dispersion of the lipid phase [135]. This contributes to a more homogeneous distribution of the active compound within the polymeric matrix and improves the film’s performance [48].

Cinnamon essential oil incorporated into chitosan films using zein–gallic acid-stabilized Pickering emulsions showed not only improved mechanical resistance but also antibacterial activity and controlled release capacity, highlighting the efficiency of the emulsion system in preserving the bioactivity of the oil [48]. These results are consistent with other studies that also used cinnamon essential oil in biopolymeric films, such as Chen et al. [8] and Ning et al. [66], which reported improvements in barrier properties, antimicrobial effectiveness, and shelf-life extension of food products. This reinforces the potential of cinnamon essential oil as a functional lipidic additive, particularly when stabilized in Pickering systems, allowing for enhanced preservation effects and compatibility with biodegradable matrices [86].

In lignin-reinforced chitosan/MCC films, lignin nanoparticles (~100 nm) improved tensile strength by 47.10%, contact angle, WVP, and UV protection, while also extending the shelf life of cherry tomatoes [142]. These results indicate that the addition of lignin nanoparticles contributes not only to structural reinforcement but also to oxidative and light stability of the packaging [153]. Both Tan et al. [148] and Li et al. [147] used chitosan as the main biopolymer matrix to develop active films reinforced with Pickering emulsions. In both cases, the incorporation of essential oils improved the barrier properties, increased antimicrobial and antioxidant activities, and successfully extended the shelf life of foods.

Incorporating *Schizochytrium limacinum* oil into chitosan films using SPI-stabilized Pickering emulsions led to a contact angle of 91.79°, WVP reduction to 1.354 × 10^−12^ g·cm/cm^2^·s·Pa, and antioxidant activity reaching 89.67%, while also demonstrating effective antibacterial action [149]. These attributes are key to improving both food preservation and packaging stability under refrigeration.

For high-fat products such as walnuts, films containing bacterial cellulose nanoparticles and cinnamon essential oil exhibited 51.24% higher tensile strength and 60–65% antioxidant/antimicrobial efficacy, delaying spoilage and oxidative degradation [8]. This highlights the potential of Pickering films in packaging applications requiring high barrier integrity and bioactivity. These results are consistent with other studies that also used cinnamon essential oil in biopolymeric films. For instance, Fan et al. [34] developed chitosan/gelatin films incorporating CEO-loaded Pickering emulsions stabilized with zein nanoparticles (average droplet size ~113 nm). Their findings demonstrated significant enhancement in both mechanical strength and barrier properties, as well as strong antimicrobial activity. Additionally, the CEO exhibited slow-release behavior, confirming the role of Pickering emulsions as an effective delivery system for bioactives in active packaging materials.

The study described by Ning et al. [66] brings a different synthesis by using a polymer matrix of alginate/konjac glucomannan and a Pickering emulsion with two active compounds together: propolis extract and tea tree essential oil. The resulting film had enhanced mechanical and barrier properties, in addition to a potent antimicrobial and antioxidant capacity, extending the shelf life of strawberries by several days. Another study also employed alginate and konjac glucomannan as the polymer matrix combined with an essential oil Pickering emulsion. Although different active compounds were used, both works demonstrated similar outcomes, showing that this formulation strategy improves tensile strength, enhances barrier properties, and provides strong antimicrobial and antioxidant activities, confirming its potential for food preservation [150].

Currently, carrageenan-based films have gained attention in the literature as promising matrices for active packaging. In the case of films incorporating oregano essential oil through Pickering emulsions, the main achievement was the stability and feasibility of incorporation, confirming the potential of this matrix for functional applications. By contrast, carrageenan/agar films containing tea tree oil and zinc sulfide nanoparticles presented a more robust formulation, with improved mechanical, barrier, and thermal stability properties, as well as moderate antioxidant and antimicrobial activity [151,152,154].

Taken together, these studies demonstrate that the performance of biopolymer films formulated with Pickering emulsions is governed by the interplay between polymer type, stabilizer, and lipid content. Chitosan-based systems dominate due to their compatibility and functional synergy with bioactives, while starch and alginate films benefit significantly from emulsion engineering to reach similar functional benchmarks.

#### 2.3.3. Liposomes as Delivery Systems in Biopolymer Films

Liposomes are nanoscale spherical vesicles, with an aqueous core, composed of one or more lipid bilayers, manufactured from surface-active substances with several intermediate HLBs and optimal curvatures close to zero, such as phospholipids [87,155,156]. Phospholipids are polar lipids, constituents of natural membranes, and their amphiphilic properties are derived from the presence of a hydrophobic tail and a hydrophilic head [157]. This amphiphilic substance allows liposomes to encapsulate hydrophobic substances in their aqueous center [158].

The electrostatic repulsion between particles depends on the level of charge and ionic strength of the medium, and its balance with the attractive van der Waals forces determines the kinetics of particle aggregation, which affects the protection and release of encapsulated compounds [159]. The formation, function, stability, behavior, and application of liposome delivery systems will mainly depend on the lipid composition, surface charge, size, and preparation method [159,160]. Liposomes possess a plurality of features and are used in various applications. Its application is generally attributed to the health area in medicines, vaccine adjuvants, and clinical applications, such as cancer treatments, antimicrobial therapy, and gene therapy [155]. However, in the food area, they represent an efficient approach for encapsulating essential oils and bioactive compounds, thereby improving their ability to disperse in water and enhancing the kinetic stability of the emulsion, which limits its destabilization and coalescence. Thus, providing a more controlled release, for example, in active packaging [161].

Andrade et al. [120] developed active films by encapsulating carvacrol in liposomes of lecithin from different origins for use in food packaging. The authors used aqueous polymeric solutions of polyvinyl alcohol (PVA), fully or partially hydrolyzed, to incorporate the liposomes into the filmogenic solutions. The hydrodynamic diameters of the liposomes varied between 179 and 294 nm, and the polydispersity index (PDI) between 0.17 and 0.36, thus proving that sonication, in general, promoted liposomes with lower diameters and PDI. Liposomes obtained with soy lecithin enriched with phosphatidylcholine (SL-PC) were the most effective in maintaining the stability of the carvacrol emulsion during film formation (DH: 195–250 nm; PDI: 0.17–0.32), which led to greater retention of carvacrol in the films, while those of sunflower lecithin (SFL) resulted in a less stable system and greater losses of carvacrol. The P-PVA was less sensitive to emulsion destabilization due to its greater binding capacity with carvacrol. Therefore, P-PVA films incorporating SL-PC liposomes loaded with carvacrol demonstrate strong potential for use in active food packaging.

**Figure 4:**
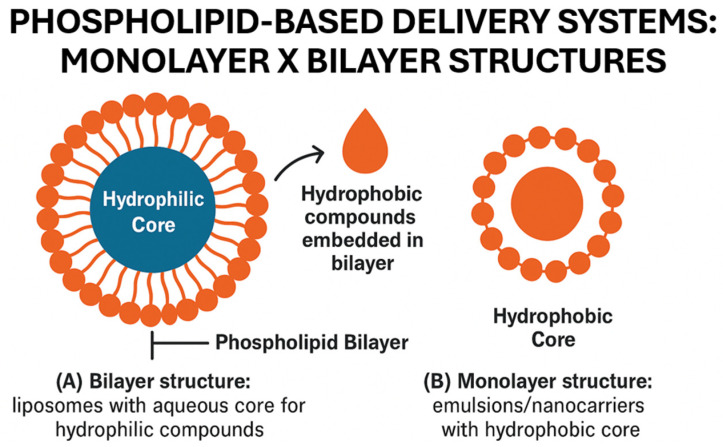
Schematic representation of liposomes as delivery systems in biopolymer films.

This behavior is illustrated in Figure 4, where various phospholipid-based delivery systems are employed to incorporate active compounds into biopolymer films. The bilayer structure, such as that of liposomes, encapsulates hydrophilic compounds in its aqueous core, while hydrophobic compounds are embedded within the phospholipid bilayer. In contrast, monolayer systems, typical of emulsions or nanocarriers, surround a hydrophobic core, making them suitable for delivering lipophilic substances. These structural differences directly influence how each system interacts with the film matrix and modulate the release of active agents over time.

## 3. Advancements in Biodegradable Packaging for Food Preservation

Biodegradable packaging has emerged as a viable and eco-friendly alternative to conventional plastics in the food system, addressing the growing demand for sustainability while preserving food. Such packaging aims to maintain food quality, safety, integrity, and shelf life while reducing the environmental burden caused by synthetic materials. This review examines recent research on biopolymer-based packaging as an active packaging material, highlighting its applications and effects on various food products during storage.

While traditional packaging provides physical barriers to protect food, the global challenge of reducing food waste and extending food shelf life has driven the development of alternative packaging technologies that are more environmentally friendly [162]. Biodegradable films composed of proteins, lipids, and polysaccharides extracted from plant tissues offer promising solutions with additional benefits for producers and consumers, reducing food waste and improper disposal [163,164].

Active packaging systems, including absorption and release mechanisms, play a crucial role in extending shelf life by capturing unwanted substances and releasing beneficial elements. For example, absorption systems use ascorbic acid and clay particles to capture oxygen and ethylene, while release systems emit elements such as antioxidants and antimicrobials [164]. The application of biodegradable packaging with Pickering emulsions in fruit preservation is highlighted, demonstrating its effectiveness in maintaining the quality and freshness of both climacteric and non-climacteric fruits while minimizing water loss and microbial contamination. Chitosan film loaded with cellulose nanocrystal-stabilized Pickering emulsions containing oleic acid has delayed superficial scald and ripening of ‘Bartlett’ pears [165].

Recent studies have demonstrated that incorporating different active biocompounds, such as anthocyanins, polyphenols, and essential oils, into biodegradable protein-based films can significantly enhance their antioxidant, antimicrobial, and barrier properties. For example, developed an innovative packaging film by crosslinking soy protein isolate and carboxymethyl chitosan with the crosslinking agent ethylene glycol, resulting in a film with excellent UV blocking (98.5%), high biodegradability, and antibacterial activity. Additionally, the authors note that the film can extend the shelf life of grapes by more than 15 days. In addition, Wang et al. [153] demonstrated the synthesis of films based on soy protein isolate reinforced with anthocyanin obtained from blueberries. The incorporation of anthocyanins not only improved the mechanical strength but also enhanced the thermal stability and water vapor barrier properties of the film. Furthermore, the film significantly extended the shelf life of edible mushrooms. Other recent studies included in this review, such as those by Zou et al. [166], Wang et al. [153], and Ren et al. [17], reinforce the multifunctionality of protein-based biodegradable films enriched with natural additives, providing insights into their pH sensitivity, antioxidant and antimicrobial properties, and the preservation of food freshness.

Table 4 presents advanced studies on the functional properties of biodegradable films composed of Pickering emulsions, where it can be observed that most of the studies described demonstrate significant improvements in the mechanical, barrier, and bioactive properties of the films, as well as in the extension of shelf life for products such as meat, fruits, and nuts. For example, films produced from chitosan and pullulan enriched with emulsions containing clove essential oil and ZnO nanoparticles showed substantial enhancements in mechanical strength and vapor and oxygen barrier properties, resulting in an extended shelf life of chicken meat by up to five days [167]. Similarly, the use of emulsions stabilized by amyloid fibrils, as reported in the study using konjac glucomannan [7], with syringa essential oil, was prepared using an encapsulation approach, achieving an encapsulation efficiency of 18.3% and a loading capacity of 11.4%. showed excellent performance in preserving cherries, prolonging their quality for approximately 10 days. In the case of γ-CD-MOF and thymol, the enhanced structural uniformity of the films contributed to a more predictable release of the kinetics of active compounds, which is crucial for consistent shelf-life extension.

Mechanical, antioxidant, antibacterial, UV [1,2,3]. These films are particularly valued for their physicochemical properties, such as tensile strength, elongation, and water vapor permeability, which can match or even surpass those of synthetic polymers under specific formulations [4,5,6].

In recent years, growing interest in multifunctional and sustainable materials. Farokhi et al. [168], in turn, presented a detailed study on gelatin-based films stabilized with chitin nanocrystals. Although the authors did not directly test the application of the films in food products, they suggest potential use for packaging fresh fruits, vegetables, and small food items such as chocolates and candies. The study also highlighted the absence of oil leakage after one year of storage, reinforcing the stability of the emulsions incorporated into the film. The combination of stabilizing particles and lipid emulsions not only enhances the physicochemical properties of the films but also expands their commercial application potential.

## 4. Industrial Challenges and Commercial Applications

While most biopolymer-based active films demonstrate excellent performance at the laboratory scale, their industrial application still faces significant technical, economic, and regulatory limitations. Among the main challenges is ensuring uniform incorporation of bioactive agents during large-scale processes such as extrusion and thermoforming, which differ considerably from laboratory methods like solvent casting or electrospinning [8].

Another key limitation lies in economic feasibility, as the high cost of certain ingredients, as well as the extraction and purification of bioactives, combined with complex processing steps, often hinders cost-effectiveness when compared to conventional plastics. Sensory issues, such as the undesirable aroma of essential oils and the migration of compounds, also persist. In addition, the lack of clear regulations and limited consumer acceptance continues to restrain broader market adoption [169,170].

Despite the challenges, promising commercial initiatives are emerging. Farokhi et al. [168] demonstrated that gelatin and chitosan-based films, incorporated with active compounds, showed no oil leakage over time and can be produced at competitive costs, representing potential alternatives for packaging vegetables and sweets. Similarly, Karunamay et al. [171] successfully applied an active film of carboxymethyl cellulose, starch, and oregano essential oil to preserve paneer, extending its shelf life for up to 12 days. Pal and Agarwal [172] developed packaging made from guar gum and beeswax capable of maintaining cheese quality for up to two months.

The growing demand for sustainable packaging suggests that this market is poised for further expansion. According to the Market Analysis Report [173], the global edible films market is projected to reach USD 4.54 billion by 2028, with a compound annual growth rate of 7.7%. This highlights the sector’s potential but also underscores the urgent need for further research focused on large-scale applications. Investments in efficient encapsulation technologies, toxicological assessments, automation, and harmonized regulatory standards are essential to enable the broader adoption of sustainable packaging within the global food supply chain.

## 5. Final Remarks

Filmogenic matrices based on proteins, polysaccharides, and carbohydrates, used as materials to produce biopolymer films, are versatile tools that can effectively serve as packaging for a wide range of foods. When adding a lipid fraction, the barrier and mechanical properties are significantly strengthened, resulting in films with superior elasticity that can be compared to synthetics, but with fewer ruptures, which expands their range of applications.

In addition, suitable results were presented in active packaging, with biodegradable films that not only have good mechanical and barrier properties but also provide improvements in the processes of oxidative degradation and microbial occurrence, through the incorporation of different encapsulation techniques of bioactive compounds in the filmogenic solution, improving delivery and increasing the shelf life of the packaged product. However, most of them are produced on a limited laboratory scale.

Large-scale production faces several challenges, including maintaining the uniformity of bioactive incorporation during industrial processing (e.g., extrusion or thermoforming), high costs of functional ingredients, and the need for regulatory standardization. Moreover, issues such as sensory acceptance, consumer perception, and the environmental impact of multi-material systems also require attention.

These advancements signify a significant shift in the development of food packaging, offering more sustainable and effective solutions for preserving food quality and extending shelf life. For instance, films incorporating blueberry anthocyanins demonstrated antioxidant activity and extended the shelf life of mushrooms by up to 4 days, while konjac glucomannan films reinforced with nitrogen-doped carbon quantum dots exhibited microbial inhibition rates of up to 99.2% and degraded completely in soil within 14 days. Future studies should prioritize the development of scalable encapsulation technologies, robust toxicological evaluations, and clear regulatory frameworks, which are essential to consolidate these sustainable materials as viable solutions for active food packaging in global supply chains.

## Figures and Tables

**Table 1:** Comparative analysis of Pickering emulsions in biopolymer films.

Bioolymeric	Compound	Emulsification Method	DS (µm)	WVP	TS	References
Fish Gelatin	Red wine pomace/Carrageenan	Casting method	0.28	0.225 g·mm/m^2^·kPah	Low in optimized film	[54,55,56]
PLA/Zein/Chitosan	Thymol essential oil	Pickering Emulsion	8.69	29.4 × 10^−6^ g m/m^2^ 24 h Pa	Increase in double-layer film	[57,58,59]
Zein	Licorice essential oil	Casting method	7–8	4.46 ± 0.178 × 10^−6^ g/Pa.dia.m	21.65 MPa	[60,61]
Zein/Pectin	Sunflower oil	Pickering Emulsion	1.15–0.6	-	-	[62,63]
Chitosan/Pectin/Starch	Rosemary and peppermint essential oil	Composite film	-	0.014 g·mm/m^2^·24 h	25.95 MPa	[59,64]
Chitosan/Sodium Alginate	Tea tree essential oil	Pickering Emulsion	3.15	1.88 × 10^−11^ kg·m/m^2^·s·Pa. min.	30.49 MPa	[65,66]
PVA	Zanthoxylum schinifolium essential oil	Pickering Emulsion	0.15	1.68 × 10^−12^ g/m·s·Pa. min	-	[67,68]
Cellulose	Oregano essential oil	Pickering Emulsion	-	24.47–37.58 g·mm/m^2^·d·kPa	1.60–2.58 MPa	[69,70]

Note: Polylactide (PLA); Droplet Size (DS); Water Vapor Permeability (WVP); (TS) Licorice (*Glycyrrhiza glabra* L.).

**Table 3:** Studies of polymer films with lipid fractions using Pickering emulsions.

Main Film/Matrix Material	Active Compound	Encapsulation Type	Droplet Size (Average)	Controlled Release	Main Improvements	References
Oxidized Corn Starch Films	Soy Protein Isolate (SPI)	Direct incorporation	N/A	Not applicable	Improvements in mechanical (Elongation from 57% to 150.93%) and hydrophobic properties (Contact Angle from 37.3° to 104.5°, WVP from 3.30 to 2.34 × 10^−12^ g·cm·cm^−2^·s^−1^·Pa^−1^).	[136,141]
Emulsions (corn oil)	Pistachio Shell CNCs (stabilizer)	Pickering Emulsions	From ~17 µm (0.1% CNC) to ~2 µm (1.5% CNC)	Implicit	Improved emulsion stability (against heat, stress, storage) and reduced droplet size.	[135]
Chitosan Films	Cinnamon Essential Oil (CEO)	Pickering Emulsion (Zein-gallic acid)	2.34 ± 0.05 µm	Yes	Increased mechanical properties and antibacterial activities of the film.	[8,48]
Chitosan/MCC/Lignin Films	Lignin Nanoparticles (LNP)	Direct incorporation of nanoparticles	LNP: ~100 nm (in optimized film)	Not applicable	Improvements in tensile strength (47.10% increase), WVP, contact angle, UV blocking; extends shelf life of cherry tomatoes.	[68,142]
Fish Gelatin Films	Palm Wax (PW)	Direct wax incorporation	N/A	Not applicable	Improved water barrier properties (WVP, solubility, swelling), tensile strength, opacity, flexibility, UV barrier.	[143,144]
Gelatin/Agar Films	ZnO-Cu Nanoparticles and Clove Essential Oil (CEO)	Pickering Emulsion (Cellulose nanofibers)	N/A	Implicit	Antioxidant and antibacterial activity; reduction in bacterial count and lipid oxidation in pork meat.	[145,146]
Chitosan/Whey Protein Isolate(WPI)/Fe^3+^ + PVA-GL	Lemon Essential Oil	Pickering Emulsion	N/A	Yes	Puncture and stretching forces of 1.03 g and 1499 g. Incorporation of essential oils improved barrier properties, strong antimicrobial and antioxidant activities. Encapsulation efficiency was over 96%, with a peak of 98.54% at 1.2% WPI concentration.	[147,148]
Chitosan	*Schizochytrium limacinum* oil	Pickering Emulsion	N/A	Yes	Increased contact angle to 91.79°, reduced WVP to 1.354 × 10^−12^ g·cm/cm^2^·s·Pa, improved UV blocking, increased antioxidant activity (89.67%), and demonstrated antibacterial activity against *S. aureus* and *E. coli*.	[34,148,149]
Sodium Alginate/Konjac Glucomannan	Propolis ethanol extract and Tea Tree Essential Oil	Pickering Emulsion	18.1 µm	Yes	Enhanced mechanical strength (TS increased by ~12.5 MPa) and barrier efficacy (WVP reduced by ~50%, UV-blocking by ~31.9%). Showed potent antimicrobial activity (inhibition zone > 18 mm) and antioxidant capacity (>80%). Extended strawberry shelf life by 3–4 days.	[66,150]
Carrageenan/agar films	Tea tree essential oil Pickering emulsion (stabilized by nanocellulose fibers) + ZnS nanoparticles	Pickering Emulsion	287.9 ± 22.5 nm (PDI 0.61)	Not applicable	ZnSNP improved tensile strength; PET slightly reduced it; combination maintained strength and improved flexibility. Enhanced WVP resistance, thermal stability, moderate antioxidant and antimicrobial activities.	[151,152]

**Table 4:** Advanced Studies on Pickering Emulsion-Based Biodegradable Films for Food Preservation.

Film Matrix	Pickering Emulsion	Properties Enhanced	Food Application/Shelf-Life	Reference
Chitosan/Pullulan	Clove essential oil with Chitosan-ZnO NPs	Mechanical, UV, O_2_ & vapor barrier, antioxidant, antibacterial	Chicken meat/+5 days	[167]
Konjac Glucomannan	Tea tree oil with Amyloid fibrils	Mechanical, barrier, antioxidant, antimicrobial	Cherries/+10 days	[7]
Gelatin	Chitin nanocrystals	Mechanical, oxygen barrier, thermal stability	Not applied	[168]
Chitosan	Bacterial cellulose with Cinnamon EO	Mechanical strength, antioxidant activity, antimicrobial activity, oxygen barrier	Walnuts/delayed spoilage and oxidative degradation	[8]

Note: Tea tree oil (*Melaleuca alternifolia*); Thymol (*Thymus vulgaris*); Clove oil (*Syzygium aromaticum*); Cinnamon EO (*Cinnamomum verum*); Litsea cubeba (*Litsea cubeba*).

## Data Availability

No new data were created or analyzed in this study.
